# Electrical impedance tomography to titrate positive end-expiratory pressure in COVID-19 acute respiratory distress syndrome

**DOI:** 10.1186/s13054-020-03414-3

**Published:** 2020-12-07

**Authors:** François Perier, Samuel Tuffet, Tommaso Maraffi, Glasiele Alcala, Marcus Victor, Anne-Fleur Haudebourg, Keyvan Razazi, Nicolas De Prost, Marcelo Amato, Guillaume Carteaux, Armand Mekontso Dessap

**Affiliations:** 1 Assistance Publique - Hôpitaux de Paris, Hôpitaux Universitaires Henri Mondor, Service de Médecine Intensive Réanimation, 51, Avenue du Maréchal de Lattre de Tassigny, 94010 Créteil Cedex, France; 2 Université Paris Est-Créteil, Faculté de Santé, Groupe de Recherche Clinique CARMAS, 94010 Créteil, France; 3 Present Address: Service de Médecine Intensive Réanimation, Centre Hospitalier Intercommunal de Créteil, 94010 Créteil, France; 4 Pulmonary Division, Cardio-Pulmonary Department, Heart Institute, University of São Paulo, São Paulo, Brazil

**Keywords:** ARDS, PEEP, Mechanical ventilation, COVID-19, Electrical impedance tomography

## Abstract

**Rationale:**

Patients with coronavirus disease-19-related acute respiratory distress syndrome (C-ARDS) could have a specific physiological phenotype as compared with those affected by ARDS from other causes (NC-ARDS).

**Objectives:**

To describe the effect of positive end-expiratory pressure (PEEP) on respiratory mechanics in C-ARDS patients in supine and prone position, and as compared to NC-ARDS. The primary endpoint was the best PEEP defined as the smallest sum of hyperdistension and collapse.

**Methods:**

Seventeen patients with moderate-to-severe C-ARDS were monitored by electrical impedance tomography (EIT) and evaluated during PEEP titration in supine (*n* = 17) and prone (*n* = 14) position and compared with 13 NC-ARDS patients investigated by EIT in our department before the COVID-19 pandemic.

**Results:**

As compared with NC-ARDS, C-ARDS exhibited a higher median best PEEP (defined using EIT as the smallest sum of hyperdistension and collapse, 12 [9, 12] vs. 9 [6, 9] cmH_2_O, *p* < 0.01), more collapse at low PEEP, and less hyperdistension at high PEEP. The median value of the best PEEP was similar in C-ARDS in supine and prone position: 12 [9, 12] vs. 12 [10, 15] cmH_2_O, *p* = 0.59. The response to PEEP was also similar in C-ARDS patients with higher vs. lower respiratory system compliance.

**Conclusion:**

An intermediate PEEP level seems appropriate in half of our C-ARDS patients. There is no solid evidence that compliance at low PEEP could predict the response to PEEP.

## Introduction

Respiratory failure is the main cause of admitting patients with COVID-19 to intensive care unit (ICU). Contrary to the classical picture of acute respiratory distress syndrome (ARDS), studies have reported many COVID-19 patients presenting with severe hypoxemia despite normal respiratory system compliance (1, 2). Two phenotypes have been suggested (3). L phenotype combined low lung weight, low elastance, and low recruitability. Hypoxemia in these patients was possibly related to impaired pulmonary perfusion, hence the theoretically limited effect of high positive end-expiratory pressure (PEEP) levels. H phenotype may combine high lung weight, high elastance, and high recruitability, which fits typical ARDS picture where standard management including relatively high PEEP could be applied. Lung physiological phenotyping of COVID-19-related ARDS (C-ARDS) is still debated, especially when compared to non-COVID-19 ARDS (NC-ARDS). Hypoxemia is a hallmark of ARDS, and positive end-expiratory pressure (PEEP) and prone position are two common tools used for its management.

The objective of this study was to describe the physiological effects of PEEP on respiratory mechanics in supine and prone position in patients who required invasive ventilation for C-ARDS and to compare it to NC-ARDS.

## Material and methods

### Patients

Patients admitted to the medical intensive care unit of Henri Mondor University Hospital for ARDS between February 27, 2019, and April 4, 2020, were included. ARDS was defined according to the Berlin definition (4). C-ARDS was confirmed by positive nasopharyngeal polymerase chain reaction for SARS-CoV-2. Patients were excluded in case of a contraindication to impedance tomography (pacemaker, implantable defibrillator, skin lesion).

### Monitoring

Patients were investigated by EIT (Enlight 1800, Timpel, Sao Paulo, Brazil). For such, a belt containing 32 electrodes was placed around the patient’s chest at the fifth or sixth intercostal space. EIT data were generated upon passing small alternate electrical current through that belt. Regional variations in impedance (∆Z) during ventilation map the tidal volume distribution in the lung and estimate regional compliance as follows. The fraction of Vt in each pixel is: V(pix) = Vt × ∆Z(pix)/∆Zglobal, and the compliance of a pixel is V(pix) divided by the global driving pressure. The PEEP titration tool helps map lung hyperdistension (regions associated with increase in local compliance when PEEP decreases) and lung collapse (regions associated with decrease in local compliance when PEEP decreases). The PEEP titration tool was used to determine the best PEEP which is defined by the best compromise between pulmonary hyperdistension and collapse.

It should be noted that compliance of the respiratory system measured by EIT may slightly differ from static compliance measured by the standard method, because plateau pressure and total PEEP are estimated on the airway pressure curve, without end-expiratory and end-inspiratory pauses.

### Protocol

Investigations were performed on deeply sedated and paralyzed patients. The tidal volume was set at 6 mL/kg of ideal body weight, the respiratory rate was adjusted to maintain normal PaCO_2,_ and the insufflation flow was set at 60 L/min. PEEP titration was performed using EIT tool, starting from a PEEP at 18 cmH_2_O (if the plateau pressure remained below 35 cmH_2_O) with a decrease of 3 cmH_2_O every two minutes, until reaching 6 cmH_2_O. No recruitment maneuver was performed before the PEEP trial. The titration began 5–10 min after setting PEEP at 18 cmH_2_O, after stabilization of the end-expiratory impedance.

In C-ARDS patients, we also performed measurement of the airway opening pressure (by insufflation at minimum flow to avoid resistive pressure) (5) and of the recruitment-to-inflation ratio (in order to determine the recruitment potential) (6). If the patient was turned prone within 24 h, the PEEP titration was repeated and then compared with the data measured in the supine position. The duration of prone positioning was 18 h. Readings of arterial blood gases (ABG) prior to exploration in supine, and last ABG at the end of proning, were collected.

If echocardiography (echo) was performed within 48 h before or after the explorations, the presence of acute cor pulmonale on standard echo or the presence of a patent foramen ovale after contrast injection was recorded. Similarly, if chest computed tomography (CT) scan was performed within 48 h before or after the explorations, the results were collected, including the presence or absence of posterior pulmonary consolidation on CT scan or pulmonary embolism on CT angiography. All CT scans were reviewed by the attending radiologist.

In all cases, at the end of the investigations, PEEP was set at the best level as evidenced by the PEEP titration tool and the respiratory mechanics.

### Statistics

Quantitative data are expressed as median [first, third quartiles]. Curves of respiratory system compliance, hyperdistention, and collapse at different PEEP levels were assessed by computing areas under the curves (AUCs), as suggested by Matthews et al. (7). Briefly, the AUC was calculated by adding the areas under the graph between each pair of consecutive observations. For the measurements *Y*15 at PEEP15 and *Y*12 at PEEP12 for example, the area between those two PEEP was the product of the PEEP difference by the average of the two measurements:

$$\begin{gathered} {\text{Area }} = \, \left( {15 \, {-} \, 12} \right) \, * \, \left( {Y15 \, + \, Y12} \right) \, / \, 2 \hfill \\ {\text{AUC}} = \frac{1}{2} \sum \left( {{\text{PEEP}}_{i + 3} - {\text{PEEP}}_{i} } \right)\left( {y_{i} + y_{i + 3} } \right) \hfill \\ \end{gathered}$$

We then compared AUCs between groups using Wilcoxon–Mann–Whitney test. Effects of prone positioning on continuous variables were studied using Wilcoxon paired test. Comparisons between C-ARDS and NC-ARDS patients relied on Mann–Whitney test for continuous variables. After determining median respiratory system compliance, patients with higher compliance values (i.e., > median) were compared with those with lower values. Owing to the exploratory nature of the study, no sample size calculation was needed.

### Ethical issues

This is an ancillary report of an ongoing prospective monocentric observational study on EIT in patients with ARDS (CPP-66/17). Written informed consent was waived due to the observational nature of the study.

## Results

### Patient characteristics and outcomes

A total of 135 ARDS patients were admitted during the study period. Among them, 105 could not be included because of a contraindication to impedance tomography [including pacemaker or implantable defibrillator (*n* = 4), skin lesion (*n* = 4)], or lack of availability of material or personnel (*n* = 97). Thus, the present study comprises 30 patients investigated by EIT with PEEP titration, including 17 with C-ARDS and 13 with NC-ARDS [bacterial pneumonia (*n* = 5), tuberculosis (*n* = 1), pneumocystis (*n* = 1), aspiration pneumonia (*n* = 3), interstitial lung disease (*n* = 1), and extra-pulmonary sepsis (*n* = 2)]. Patients were explored a median of 1 [1, 2] days after intubation. The characteristics and outcomes of included patients are summarized in Table 1. C-ARDS and NC-ARDS patients had similar characteristics and outcomes, except for significantly lower SAPS 2 at admission, and more cor pulmonale on echocardiography in the former group.

**Table 1:** Characteristics of 30 patients with acute respiratory distress syndrome induced or not by coronavirus disease-19

	C-ARDS (*n* = 17)	NC-ARDS (*n* = 13)	*p* value
Patients' characteristics			
Age (years)	54 [50, 67]	69 [53, 71]	0.15
Male, *n* (%)	16/17 (94%)	11/13 (85%)	0.56
Weight (kg)	90 [80, 106]	80 [66, 90]	0.07
Body mass index, kg/m^2^	30.2 [27.8, 33.2]	28.7 [24.8, 31.1]	0.16
History of COPD, *n* (%)	1/17 (6%)	1/13 (8%)	> 0.99
History of chronic heart failure, *n* (%)	3/17 (18%)	4/13 (31%)	0.67
History of chronic kidney failure, *n* (%)	3/17 (18%)	3/13 (23%)	> 0.99
Immunosuppression, *n* (%)	1/17 (6%)	2/13 (15%)	0.56
Time from first symptoms to intubation (days)	9 [6.5, 10]	NA	
SAPS 2	34 [27, 38]	62 [42, 83]	0.01
PaO_2_/FiO_2_ at intubation (mmHg)	98 [90, 144]	135 [80, 182]	0.64
CT scan and echocardiography			
Presence of lung consolidation on CT scan, *n* (%)	2/11 (18%)	4/5 (80%)	0.04
Pulmonary embolism on CT angiography, *n* (%)	3/8 (38%)	1/5 (20%)	> 0.99
Acute cor pulmonale on echocardiography, *n* (%)	8/17 (47%)	1/13 (8%)	0.04
Patent foramen ovale, *n* (%)	1/17 (6%)	0	> 0.99
Outcomes			
Need for vasopressor, *n* (%)	11/17 (65%)	11/13 (85%)	0.41
ECMO upon ICU stay, *n* (%)	2/17 (12%)	1/13 (8%)	> 0.99
Tracheotomy during ICU stay, *n* (%)	3/17 (18%)	0	0.24
Duration of mechanical ventilation (days)	13 [9, 29]	13 [8, 22]	0.53
Duration of ICU stay (days)	18 [12, 30]	17 [11, 22]	0.75
Death in ICU, *n* (%)	4/17 (24%)	6 /13 (46%)	0.26

Continuous variables are expressed as median [interquartile range]

*C-ARDS* coronavirus disease-19-related acute respiratory distress syndrome, *NC-ARDS* noncoronavirus disease-19-related acute respiratory distress syndrome, *BMI* body mass index, *COPD* chronic obstructive pulmonary disease, *SAPS* simplified acute physiology score, *CT* computed tomography, *ECMO* extracorporeal membrane oxygenation, *ICU* intensive care unit, *NA* not available

### Respiratory mechanics and PEEP titration

**Fig. 1:**
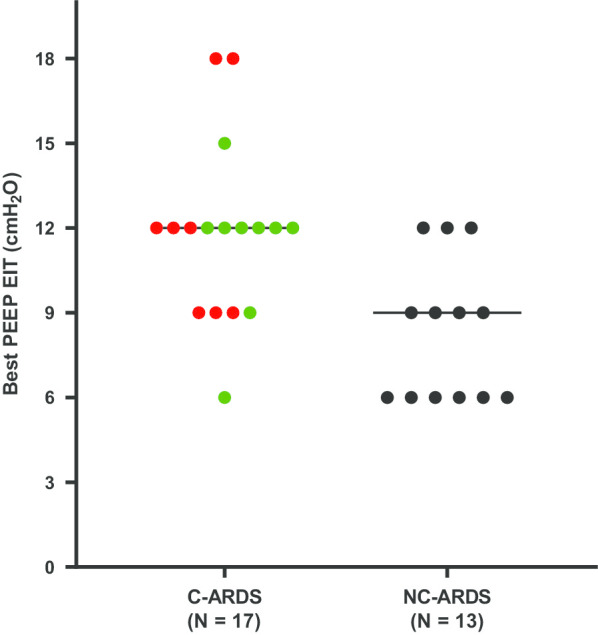
Best positive end-expiratory pressure as defined by electric impedance tomography in patients with acute respiratory distress syndrome related to coronavirus disease-19 (**a**) or not (**b**), with lower (red circles) versus higher (green circles) respiratory system compliance

**Fig. 2:**
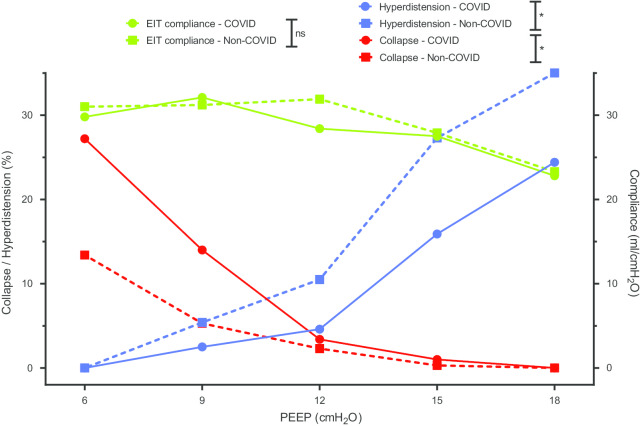
Positive end-expiratory pressure titration in patients with acute respiratory distress syndrome associated or not with coronavirus disease-19. * denotes a *p* value < 0.05 for Wilcoxon–Mann–Whitney test comparing area under the curve.

C-ARDS and NC-ARDS patients were similar in terms of hypoxemia, but with a trend to have higher body weights and respiratory system compliance in the former group (Tables 1, 2). The best PEEP (defined using EIT as the smallest sum of hyperdistension and collapse) ranged from 6 to 18 cmH_2_O, with a higher value in C-ARDS than NC-ARDS (Table 2, Fig. 1). C-ARDS patients had more derecruitment at lower PEEP and less hyperdistension at higher PEEP as compared to NC-ARDS patients (Table 2; Fig. 2).

**Table 2:** Comparison of respiratory mechanics and PEEP titration in supine position, for patients with acute respiratory distress syndrome induced or not by coronavirus disease-19

	C-ARDS (*n* = 17)	NC-ARDS (*n* = 13)	*p* value
Respiratory mechanics			
Respiratory rate (breath/min)	30 [28, 35]	31 [29, 31]	0.74
Tidal volume (ml/kg of PBW)	6.1 [5.9, 6.3]	6.2 [6.0, 6.3]	0.58
C_RS_ at low PEEP (mL/cmH_2_O)	40 [32, 47]	35 [26, 38]	0.07
Airway opening pressure (cmH_2_O)	2 [0, 4]	NA	
Recruitment-to-inflation ratio	0.46 [0.33, 0.52]	NA	
PaO_2_/FiO_2_ before EIT explorations (mmHg)	133 [96, 180]	120 [110, 137]	0.40
EIT-PEEP titration			
Best PEEP^a^ (cmH_2_O)	12 [9, 12]	9 [6, 9]	< 0.01
Hyperdistension at PEEP 18 cmH_2_O, %	24 [17, 30]	35 [25, 41]	0.02
AUC for hyperdistension	111 [68, 136]	193 [119, 226]	0.03
Collapse at PEEP 6 cmH_2_O, %	27 [20, 35]	13 [7, 19]	< 0.01
AUC for collapse	94 [59, 141]	45 [31, 61]	< 0.01

^a^Defined using EIT as the smallest sum of hyperdistension and collapse. Continuous variables are expressed as median [interquartile range]

*C-ARDS* coronavirus disease-19-related acute respiratory distress syndrome, *NC-ARDS* noncoronavirus disease-19-related acute respiratory distress syndrome, *PBW* predicted body weight, *C*_*RS*_ respiratory system compliance, *PEEP* positive end-expiratory pressure, *EIT* electrical impedance tomography, *AUC* area under the curve, *NA* not available

### Effect of respiratory system compliance and proning in C-ARDS patients

**Fig. 3:**
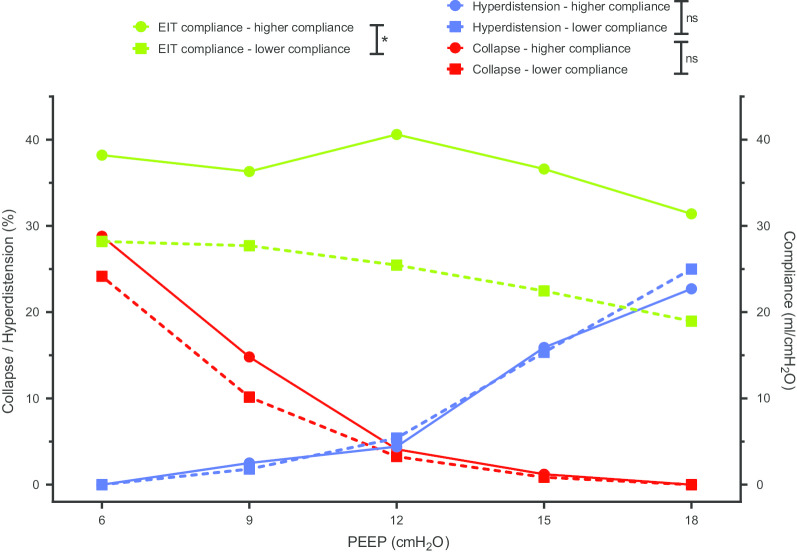
Positive end-expiratory pressure titration in patients with coronavirus disease-19-related acute respiratory distress syndrome, with lower versus higher respiratory system compliance. * denotes a *p* value < 0.05 for Wilcoxon–Mann–Whitney test comparing area under the curve

We also compared two C-ARDS patient subgroups based on the compliance of the respiratory system at low PEEP (Table 3, Fig. 3). Body mass index, time from first symptoms to exploration, recruitment-to-inflation ratio, and PaO_2_/FiO_2_ were similar in C-ARDS patients with lower versus higher respiratory system compliance. The response to PEEP was similar in the two subgroups in terms of collapse, hyperdistension, or best PEEP (Table 3, Fig. 3). There was no correlation between recruitment-to-inflation ratio and best PEEP (rho = − 0.37, *p* = 0.14).

**Table 3:** Comparison of patients with coronavirus disease-19-related acute respiratory distress syndrome associated with lower versus higher respiratory system compliance

	Compliance ≥ 40 mL/cmH_2_O (*n* = 9)	Compliance < 40 mL/cmH_2_O (*n* = 8)	*p* value
Body mass index (kg/m^2^)	29.8 [27.9, 33.1]	30.3 [24.4, 33.4]	0.67
Time from first symptoms to exploration (days)	9 [6.5, 12]	10 [9, 11]	0.63
PaO_2_/FiO_2_ before EIT (mmHg)	138 [89, 182]	130 [118, 150]	0.96
Respiratory system compliance (mL/cmH_2_O)	47 [46, 52]	31.5 [29, 37]	< 0.01
Recruitment-to-inflation ratio	0.48 [0.41, 0.62]	0.4 [0.31, 0.49]	0.25
Increase in PaO_2_/FiO_2_ after proning (mmHg)	52.5 [38, 91]	52 [12, 68]	0.53
Duration of proning (h)	18.5 [18, 21]	18 [17.5, 20.5]	0.87
EIT-PEEP titration			
Best PEEP^a^ (cmH_2_O)	12 [12]	12 [9, 13.5]	0.92
AUC for compliance	444 [345, 513]	293 [260, 316]	< 0.01
Hyperdistension at PEEP 18 (%)	23 [17, 31]	25 [18, 27]	0.70
AUC for hyperdistension	111 [68, 141]	108 [78, 131]	0.81
Collapse at PEEP 6 (%)	29 [21, 39]	24 [19 – 31]	0.61
AUC for collapse	104 [59, 141]	78 [66, 115]	0.74

^a^Defined using EIT as the smallest sum of hyperdistension and collapse. Continuous variables are expressed as median [interquartile range]

*PEEP* positive end-expiratory pressure, *EIT* electrical impedance tomography, *AUC* area under the curve

**Fig. 4:**
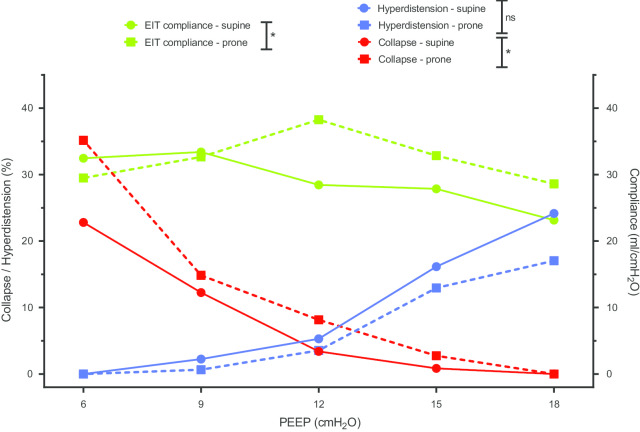
Positive end-expiratory pressure titration in patients with coronavirus disease-19-related acute respiratory distress syndrome, in supine versus prone position. * denotes a *p* value < 0.05 for Wilcoxon–Mann–Whitney test comparing area under the curve

In C-ARDS patients, proning was resulted in higher values of EIT-measured respiratory system compliance and more collapse at lower PEEP as compared to supine position, while hyperdistension and best PEEP did not significantly change (Table 4, Fig. 4). The median delay between prone positioning and PEEP titration was 4 [2, 11] h, depending on the availability of EIT device and investigators.

**Table 4:** Comparison of PEEP titration in supine versus prone position, for patients with coronavirus disease-19-related acute respiratory distress syndrome

	C-ARDS in supine position (*n* = 14)	C-ARDS in prone position (*n* = 14)	*p* value
PaO_2_/FiO_2_ (mmHg)	107 [93, 136]	179 [127, 190]	< 0.01
EIT-PEEP titration			
Best PEEP^a^ (cmH_2_O)	12 [9, 12]	12 [10, 15]	0.59
AUC for compliance	344 [316, 439]	455 [332, 494]	0.04
Hyperdistension at PEEP 18 cm H_2_O (%)	24 [17, 30]	17 [15, 41]	0.63
AUC for hyperdistension	113 [68, 134]	80 [40, 194]	0.82
Collapse at PEEP 6 cm H_2_O (%)	23 [19, 37]	35 [23, 42]	0.07
AUC for collapse	86 [58, 139]	173 [135, 261]	< 0.01

^a^Defined using EIT as the smallest sum of hyperdistension and collapse. Continuous variables are expressed as median [interquartile range]

*PEEP* positive end-expiratory pressure, *EIT* electrical impedance tomography, *AUC* area under the curve in supine and prone position (see Fig. 4)

## Discussion

The main findings of our report are as follows: (1) the median value of the best PEEP defined using EIT as the smallest sum of hyperdistension and collapse was 12 cmH_2_O in C-ARDS patients in prone and supine position; (2) baseline respiratory system compliance was not predictive of the response to PEEP; and (3) compared with NC-ARDS, C-ARDS exhibited more collapse at low PEEP and less hyperdistension at high PEEP.

### PEEP level setting

In our study, an intermediate PEEP level of around 12 cmH_2_O seemed appropriate in more than half of C-ARDS patients. A lower PEEP level was associated with significant alveolar collapse. Another study reporting PEEP titrations by EIT in 15 C-ARDS patients showed almost 50% collapse at low PEEP (8). In our work, this collapse was worse than that observed in NC-ARDS patients. This finding could be explained by a higher prevalence of obesity or overweight in COVID-19 patients (9); our results showed a trend toward higher body weight in the C-ARDS group. On the other hand, this collapse does not seem to be completely explained by higher airway opening pressures in C-ARDS patients as only two out of 17 patients had an airway opening pressure greater than 5 cmH_2_O.

A higher PEEP level was associated with hyperdistension, and only three patients had an ideal PEEP level (defined by the EIT PEEP titration tool) greater than 12 cmH_2_O. These findings are aligned with those from another study in C-ARDS (10), which showed a decrease in compliance and an increase in dead volume at higher PEEP, indicating hyperdistension and absence of recruitment (11). The benefit of higher PEEP should also be weighed against hemodynamic tolerance since the incidence of pulmonary thrombosis is high in C-ARDS patients (12,13) and half of our patients exhibited cor pulmonale. Overall, our results are consistent with recent data suggesting a variable potential for recruitment in C-ARDS patients (14–16).

### Prone position

In the prone position, the best PEEP was not significantly different from the supine position. Previous work in NC-ARDS found consistent results. Cornejo and al (17), using CT scan, showed that the percentage of recruitment was similar in both positions (36% in supine et 32% in prone) when PEEP increased from 5 to 15 cmH_2_O; Aguirre-Bermeo and al (18), using the nitrogen washout/washin technique, showed that PEEP-induced lung volume recruitment did not significantly change in prone versus supine position. The higher collapse at lower PEEP in prone versus supine may be explained by the local increase in ventral chest wall elastance in the former position. However, the benefit of proning in this situation may be explained by the persistence of a predominantly dorsal perfusion in prone (19).

Only six NC-ARDS patients were turned prone within 48 h of exploration in supine position, and these patients did not have a PEEP titration in prone position. Therefore, we were unable to compare PEEP titrations in prone between C-ARDS and NC-ARDS.

### Respiratory system compliance

Some authors have described a significant proportion of C-ARDS patients ventilated with normal compliance, with a median value around 50 mL/cmH_2_O (2). They distinguished two profiles: low recruiters characterized by low elastance, low lung weight, and a priori little benefit from higher PEEP and prone positioning; and high recruiters characterized by high elastance, high lung weight, and possible response to higher PEEP and prone positioning. However, since then, several studies have shown a significant and early alteration of compliance in C-ARDS (16,20,21). Furthermore, in large cohort studies, compliance of C-ARDS patients was either slightly higher (22) or similar (23) to compliance of NC-ARDS patients.

Prior to COVID-19 pandemic, many studies sought to determine factors predicting the response to PEEP or proning. In NC-ARDS, neither compliance at low PEEP nor CT-scan radiological pattern was predictive of recruitment at high PEEP (11) or improvement in oxygenation in the prone position (24). In the present study, we found a comparable response to PEEP in patients with higher versus those with lower respiratory system compliance. Similarly, after prone positioning, the increase in PaO_2_/FiO_2_ ratio was comparable in both subgroups. Their numbers (eight to nine patients per subgroups) were certainly low, and the CT-scan profile was not taken into account. However, there is no evidence that the sole measurement of the respiratory system compliance can predict the response to PEEP or proning in C-ARDS patients. Similarly, the recruitment-to-inflation ratio did not correlate with the best PEEP. Therefore, different levels of PEEP should probably be tested regardless of baseline compliance in order to adjust ventilator settings to individual needs.

### Strengths and limitations

The strengths of our study rely on the comprehensive physiological assessment and the comparison with NC-ARDS. To our knowledge, this is the first study to compare the PEEP response of C-ARDS and NC-ARDS in supine and prone position, using EIT. The main limitation of our work is the sample size. Patients’ enrollment was greatly impacted by the heavy workload upon COVID pandemic and the lack of time to set up monitoring and conduct investigations. Second, it was not possible to perform chest CT scan in all patients at the time of explorations. As a result, we were neither able to correlate the CT-scan results with physiological findings nor to evaluate the effect of PEEP according to the radiological phenotype. Third, we were unable to compare PEEP titrations in prone between C-ARDS and NC-ARDS.

## Conclusion

Our study characterized the lung physiology of C-ARDS with the following findings. An intermediate PEEP seemed appropriate to minimize collapse and hyperdistension in more than half of our C-ARDS patients in supine or prone position. Compliance at low PEEP alone could not predict the response to PEEP.

## Supplementary Information

**Additional file 1**. Median and interquartile values of hyperdistension, collapse and EIT-compliance for each level of PEEP during PEEP titration in: S1) Patients with NC-ARDS versus C-ARDS; S2) C-ARDS patients with lower versus higher respiratory system compliance; S3) C-ARDS patients in supine versus prone position.

## Data Availability

The datasets used and/or analyzed during the current study are available from the corresponding author on reasonable request.

## Abbreviations

C-ARDS: Coronavirus disease-19-related acute respiratory distress syndrome
NC-ARDS: Noncoronavirus disease-19-related acute respiratory distress syndrome
PEEP: Positive end-expiratory pressure
EIT: Electrical impedance tomography
ICU: Intensive care unit
ABG: Arterial blood gases
CT: Computed tomography
AUC: Area under the curve
