# Supplementary material for: Electrical impedance tomography to titrate positive end-expiratory pressure in COVID-19 acute respiratory distress syndrome
Source: Crit Care. 2020 Dec 7;24:678. doi: 10.1186/s13054-020-03414-3 (PMC7719729; doi:10.1186/s13054-020-03414-3)
Supplement: Supplementary file 1 — Additional file 1. Median and interquartile values of hyperdistension, collapse and EIT-compliance for each level of PEEP during PEEP titration in: S1) Patients with NC-ARDS versus C-ARDS; S2) C-ARDS patients with lower versus higher respiratory system compliance; S3) C-ARDS patients in supine versus prone position. [file 13054_2020_3414_MOESM1_ESM.docx]

**S1: PEEP titration in C-ARDS versus NC-ARDS**

|  |  | **PEEP 6** | **PEEP 9** | **PEEP 12** | **PEEP 15** | **PEEP 18** |
| --- | --- | --- | --- | --- | --- | --- |
| **Hyperdistension** | C-ARDS | 0 [0, 0] | 3 [0, 4] | 5 [3, 10] | 16 [12, 18] | 24 [17, 30] |
|  | NC-ARDS | 0 [0, 0] | 5 [1, 9] | 11 [7, 20] | 27 [17, 32] | 35 [25, 41] |
| **Collapse** | C-ARDS | 27 [20, 35] | 14 [8, 23] | 3 [3, 5] | 1 [0, 2] | 0 [0, 0] |
|  | NC-ARDS | 13 [7, 19] | 5 [5, 9] | 2 [1, 4] | 0 [0, 1] | 0 [0, 0] |
| **EIT-compliance** | C-ARDS | 30 [28, 38] | 32 [28, 36] | 28 [27, 41] | 28 [22, 37] | 23 [19, 31] |
|  | NC-ARDS | 31 [26, 35] | 31 [24, 37] | 32 [22, 34] | 28 [18, 32] | 23 [18, 30] |

**S2: PEEP titration in C6ARDS with lower versus higher respiratory system compliance**

|  |  | **PEEP 6** | **PEEP 9** | **PEEP 12** | **PEEP 15** | **PEEP 18** |
| --- | --- | --- | --- | --- | --- | --- |
| **Hyperdistension** | Higher-compliance | 0 [0, 0] | 3 [1, 4] | 4 [4, 10] | 16 [10, 18] | 23 [17, 31] |
|  | Lower-compliance | 0 [0, 0] | 2 [0, 3] | 5 [3, 10] | 15 [13, 18] | 25 [18, 27] |
| **Collapse** | Higher-compliance | 29 [21, 39] | 15 [9, 23] | 4 [3, 5] | 1 [1, 2] | 0 [0, 0] |
|  | Lower-compliance | 24 [19, 31] | 10 [8, 16] | 3 [3, 5] | 1 [0, 2] | 0 [0, 0] |
| **EIT-compliance** | Higher-compliance | 38 [35, 41] | 36 [35, 44] | 41 [29, 45] | 37 [28, 44] | 31 [24, 39] |
|  | Lower-compliance | 28 [26, 29] | 28 [23, 29] | 25 [22, 27] | 22 [19, 25] | 19 [17, 21] |

**S3: PEEP titration in C-ARDS in supine versus prone position**

|  |  | **PEEP 6** | **PEEP 9** | **PEEP 12** | **PEEP 15** | **PEEP 18** |
| --- | --- | --- | --- | --- | --- | --- |
| **Hyperdistension** | Supine | 0 [0, 0] | 2 [0, 4] | 5 [3, 10] | 16 [11, 18] | 24 [17, 30] |
|  | Prone | 0 [0, 0] | 1 [0, 2] | 4 [1, 15] | 13 [4, 29] | 17 [15, 41] |
| **Collapse** | Supine | 23 [19, 37] | 12 [8, 22] | 3 [3, 5] | 1 [0, 2] | 0 [0, 0] |
|  | Prone | 35 [23, 42] | 15 [11, 30] | 8 [5, 14] | 3 [1, 5] | 0 [0, 0] |
| **EIT-compliance** | Supine | 32 [28, 38] | 33 [29, 36] | 28 [27, 40] | 28 [24, 36] | 23 [20, 31] |
|  | Prone | 30 [23, 35] | 33 [28, 38] | 38 [27, 41] | 33 [25, 39] | 29 [23, 34] |

*Continuous variables are expressed as median [interquartile range].*

*Definition of abbreviations: C-ARDS: coronavirus disease 19 related acute respiratory distress syndrome; NC-ARDS: non-coronavirus disease 19 related acute respiratory distress syndrome; EIT: electrical impedance tomography; PEEP: positive end-expiratory pressure.*
